# TGF-β/Smad3 Stimulates Stem Cell/Developmental Gene Expression and Vascular Smooth Muscle Cell De-Differentiation

**DOI:** 10.1371/journal.pone.0093995

**Published:** 2014-04-09

**Authors:** Xudong Shi, Daniel DiRenzo, Lian-Wang Guo, Sarah R. Franco, Bowen Wang, Stephen Seedial, K. Craig Kent

**Affiliations:** Department of Surgery, University of Wisconsin Hospital and Clinics, Madison, Wisconsin, United States of America; William Harvey Research Institute, Barts and The London School of Medicine and Dentistry, Queen Mary University of London, United Kingdom

## Abstract

Atherosclerotic-associated diseases are the leading cause of death in the United States. Despite recent progress, interventional treatments for atherosclerosis can be complicated by restenosis resulting from neo-intimal hyperplasia. We have previously demonstrated that TGF-β and its downstream signaling protein Smad3∶1) are up-regulated following vascular injury, 2) together drive smooth muscle cell (SMC) proliferation and migration and 3) enhance the development of intimal hyperplasia. In order to determine a mechanism through which TGF-β/Smad3 promote these effects, Affymetrix gene expression arrays were performed on primary rat SMCs infected with Smad3 and stimulated with TGF-β or infected with GFP alone. More than 200 genes were differentially expressed (>2.0 fold change, p<0.05) in TGF-β/Smad3 stimulated SMCs. We then performed GO term enrichment analysis using the DAVID bioinformatics database and found that TGF-β/Smad3 activated the expression of multiple genes related to either development or cell differentiation, several of which have been shown to be associated with multipotent stem or progenitor cells. Quantitative real-time PCR confirmed up-regulation of several developmental genes including FGF1, NGF, and Wnt11 (by 2.5, 6 and 7 fold, respectively) as well as stem/progenitor cell associated genes CD34 and CXCR4 (by 10 and 45 fold, respectively). In addition, up-regulation of these factors at protein levels were also confirmed by Western blotting, or by immunocytochemistry (performed for CXCR4 and NGF). Finally, TGF-β/Smad3 down regulated transcription of SMC contractile genes as well as protein production of smooth muscle alpha actin, calponin, and smooth muscle myosin heavy chain. These combined results suggest that TGF-β/Smad3 stimulation drives SMCs to a phenotypically altered state of de-differentiation through the up-regulation of developmental related genes.

## Introduction

Cardiovascular disease is the leading cause of death and a major cause of disability in this country. Each year over a million vascular reconstructions are performed to restore circulation to ischemic organs. Unfortunately, a good number of these eventually fail due to intimal hyperplasia which re-narrows the vessel lumen. Currently, the only clinical approach to prevent restenosis is the drug eluting stent, which is implanted into a vessel following angioplasty, releasing drugs such as rapamycin or paclitaxel. Even with the use of these stents, the incidence of restenosis remains ∼15% in the coronary arteries and higher in the peripheral circulation [1]. Moreover, patients are predisposed to a risk of thrombosis resulting in sudden death. Therefore, it is imperative to further delineate the underlying mechanisms of restenosis with the goal of devising better strategies for prevention.

Intimal hyperplasia, or injury-triggered growth of the subintimal layer of an artery or vein is the primary pathological event leading to restenosis. Essential to this process is the transformation of medial smooth muscle cells (SMCs) from a quiescent and contractile state to a synthetic phenotype, active in the production of extracellular matrix [2]. These “activated” cells quickly multiply and migrate into the subintimal space forming a highly cellular neointimal plaque. While attracting enormous interest, the nature of this SMC phenotypic transformation has remained controversial. A common observation is that injury-stimulated SMCs re-gain the ability to proliferate and migrate while losing signature SMC characteristics, *i.e.* loss of the spindle morphology and a decrease in SMC markers such as smooth muscle actin (SMA), calponin, and myosin heavy chain (MHC). It has been hypothesized by some that SMCs, in response to injury, switch to a phenotypically modulated state [2]. However, in a recent study, it was hypothesized that this “synthetic” cell population is derived from resident stem cells in the media, rather than de-differentiation of mature medial SMCs [3].

TGF-β is known to be a regulator of a great number of cell types. Following ligand binding, the TGF-β receptor heterodimer is activated and phosphorylates the two cognate Smads, Smad2 and Smad3, which then bind to Smad4. The resulting complex translocates to the nucleus and regulates the expression of a plethora of genes by binding to their promoters [4]. The regulatory power of Smad3 as a master transcription factor is augmented or modulated by interactions with some 50 co-transcription factors. Aside from these canonical pathways, TGF-β is also able to regulate gene expression through non-canonical pathways, typically MAPK including ERK, JNK, and p38. Moreover, these noncanonical pathways crosstalk with the canonical TGF-β/Smad3 pathway forming a complex TGF-β signaling network. As such, TGF-β function can vary and depends upon cell type, the extracellular environment, as well as the signaling context [4].

TGF-β has been found to be an inhibitor of proliferation in many cell types including cancer cells. In vascular SMCs we and several other groups have observed *in vitro*, that TGF-β inhibits cell proliferation as well as migration [5], [6], [7]. This is surprising in that TGF-β has been found to enhance the formation of intimal hyperplasia at the time of arterial injury, which is characterized by SMC proliferation and migration [8]. Our group has found that the anti-proliferative effect of TGF-β is reversed in the presence of elevated Smad3 expression, and Smad3 expression is enhanced following arterial injury. Moreover, adenoviral expression of Smad3 following arterial injury exacerbates the formation of intimal hyperplasia [6]. Thus, in a background of elevated Smad3, the function of TGF-β in SMCs is paradoxically opposite of the anti-proliferative effect generally observed in cell culture. We reason that when Smad3 protein is increased (*in vitro* or *in vivo*), the canonical TGF-β/Smad3 pathway may over-ride other TGF-β-associated pathways and dominate the TGF-β signaling network, leading to an altered outcome in SMC behavior. We hypothesize that TGF-β/Smad3 signaling alters the expression profile of the Smad3-responsive genome thus driving SMCs to a distinct, de-differentiated fate.

In order to investigate the TGF-β/Smad3-directed SMC transcriptome, we performed gene array following TGF-β stimulation of primary rat aortic SMCs transduced with an adenovirus expressing Smad3, mimicking the *in vivo* condition of TGF-β and Smad3 elevation following arterial injury. Interestingly, our data reveal, in response to TGF-β/Smad3, up-regulation of a large number of genes associated with development and/or multipotent stem cells or progenitor cells. Our findings suggest a novel mechanism whereby elevated TGF-β/Smad3 reverses the developmental lineage to a de-differentiated state. These findings provide novel, important insight into the disparate function of TGF-β *in vitro* and *in vivo* as well as the molecular mechanisms of intimal hyperplasia.

## Materials and Methods

### Ethics Statement

The experiments involving animal use were carried out in strict accordance with the recommendations in the Guide for the Care and Use of Laboratory Animals of the National Institutes of Health. The protocol (Permit Number: M02273) was approved by the Institutional Animal Care and Use Committee (IACUC) of the University of Wisconsin-Madison. All surgery was performed under isoflurane anesthesia, and all efforts were made to minimize suffering.

### Reagents

Recombinant human TGF-β1 was purchased from R&D Systems (Minneapolis, MN). Dulbecco’s modified Eagle’s medium (DMEM) and cell culture reagents were from Invitrogen (Carlsbad, CA). All the other reagents were from Sigma (St. Louis MO) unless otherwise specified.

### Smooth Muscle Cell Culture and Viral Infection

Rat aortic vascular smooth muscle cells (SMCs) were isolated from the thoracoabdominal aorta of male Sprague-Dawley rats based on a protocol described by Clowes *et al*. [9]. Vascular SMCs were used at passages 5 to 6 for all experiments and were maintained in DMEM supplemented with 10% fetal bovine solution (FBS) at 37°C with 5% CO_2_. For experiments involving Smad3 overexpression, vascular SMCs were infected with adenovirus (3×10^4^ particles/cell) expressing Smad3 (or GFP control) in DMEM containing 2% FBS for 4 h at 37°C followed by recovery in media containing 10% FBS. After overnight incubation, cells were starved in DMEM containing 0.5% FBS for 24 h. The cells were then treated with recombinant TGF-β (5 ng/ml) or solvent for desired hours. For microarray analysis, passage 5 SMCs from 3 different animals were infected with AdSmad3 or AdGFP control followed by treatment with TGF-β (5 ng/ml) or solvent (for GFP control) for 24h. Adenoviral vectors expressing Smad3 (AdSmad3) and GFP (AdGFP) were constructed as previously described [7].

### RNA Isolation and Quality Control

Total RNA from SMCs treated with AdSmad3 and TGF-β or AdGFP alone was isolated using the Qiagen RNA/DNA Mini Kit (Qiagen, Gaithersburg, MD) following the manufacturer’s instructions. For quality control, 1 μg of total RNA was analyzed by capillary electrophoresis using the RNA 6000 Nano LabChip and the Agilent Bioanalyzer 2100 (Agilent Technologies, Palo Alto, CA). All RNA samples used in this study showed no sign of degradation.

### Microarray

Microarray was carried out at the University of Wisconsin-Madison Biotechnology Center (Madison, WI). Whole transcript target labeling was carried out with total RNA using Ambion’s expression kit to generate terminal labeled cDNA (Austin, TX). Labeled cDNA was hybridized to rat Genechip (Rat Gene 1.0 ST Array) overnight in the AFX HybOven480 (Santa Clara, CA), and then post-processed on the Fluidics450 station. Post-hybridization washing was performed according to manufacturer’s instructions. The microarray slides were scanned on a G7 scanner (Affymetrix, Santa Clara, CA). Data were extracted using the Affyrmetrix console software. The complete data set is available at Gene Expression Omnibus (GEO Accession: GSE54624).

### Basic Analyses

The fold changes of gene expression, P values and heat maps were calculated using the ArrayStar software (DNASTAR, Inc., Madison, WI). A moderated t-test was performed to identify putative genes with significant expression changes. Putative genes that were up or down regulated more than 2-fold with a P value less than 0.05 were considered as differentially expressed and selected for further analysis.

### Functional Analysis of Significantly Affected Genes

Genes that were up or down regulated more than 2 fold (P<0.05) after TGF-β/Smad3 treatment compared to GFP control were submitted to David Bioinformatics Resources 6.7, NIAID/NIH (http://david.abcc.ncifcrf.gov/). The overall functions regulated by these TGF-β/Smad3 modulated genes were identified by Functional Annotation Clustering and ranked by enrichment scores.

### Real-time Quantitative PCR (qRT-PCR)

mRNA was isolated using RNeasy Plus Mini Kit (Qiagen, Valencia, CA). Potential contaminating genomic DNA was removed by using gDNA Eliminator columns provided in the kit. The purified mRNA (2 μg) was used for the first-strand cDNA synthesis and quantitative RT-PCR was performed using the 7500 Fast Real-Time PCR System (Applied Biosystems, Carlsbad, CA). Each cDNA template was amplified in triplicates using SYBR Green PCR Master Mix (Applied Biosystems, Carlsbad, CA) with gene specific primers.

### Western Blotting Analysis

Cells were lysed in RIPA buffer (50 mM Tris, 150 mM NaCl, 1% Nonidet P-40 and 0.1% sodium dodecyl sulfate) containing Protease Inhibitor Cocktail I (Millipore,Billerica, MA). Protein concentration was determined by Bio-Rad DC Protein Assay kit (Hercules, CA). Thirty micrograms of proteins from each sample were separated by 10% SDS-PAGE and transferred to nitrocellulose membranes. Protein levels were assessed by immunoblotting with the following rabbit antibodies for FGF-1, NGF, Wnt11, CXCR4, CD34 (Santa Cruz Biotechnology, Santa Cruz, CA), calponin, and myosin heavy chain (Abcam, Cambridge, MA), mouse antibodies for α-smooth muscle actin and β-actin (Sigma, St. Louis, MO). After incubation with appropriate primary antibodies and then horseradish peroxidase-conjugated secondary antibodies, the specific protein bands on the membranes were visualized by using enhanced chemiluminescence reagents (Pierce, Davenport, IL).

### Immunocytochemistry

Immunostaining was performed to detect *in situ* the expression of CXCR4 and NGF in SMCs, following our published method. Cells were prepared by fixation in 4% paraformaldehyde in PBS for 10 minutes. The cells were then permeabilized in 0.1% triton x-100 in PBS and blocked for 1 h in 5% BSA/5% donkey serum for 1 h. Primary antibody was applied overnight at 4°C, and then a secondary antibody was incubated with the cells for 1 h at room temperature followed by fluorescence microscopy.

### Statistical Analysis

Statistical analysis of the data other than microarray results was conducted using a one–way analysis of variance (ANOVA) and data were presented as mean ± SEM derived from at least three independent experiments, unless otherwise stated. If significant, the ANOVA was followed by Tukey’s multiple comparison test. P value less than 0.05 was regarded as statistically significant.

## Results

### Stimulation of Vascular SMCs with TGF-β/Smad3 Drives Large-scale Gene Expression

**Figure 1:**
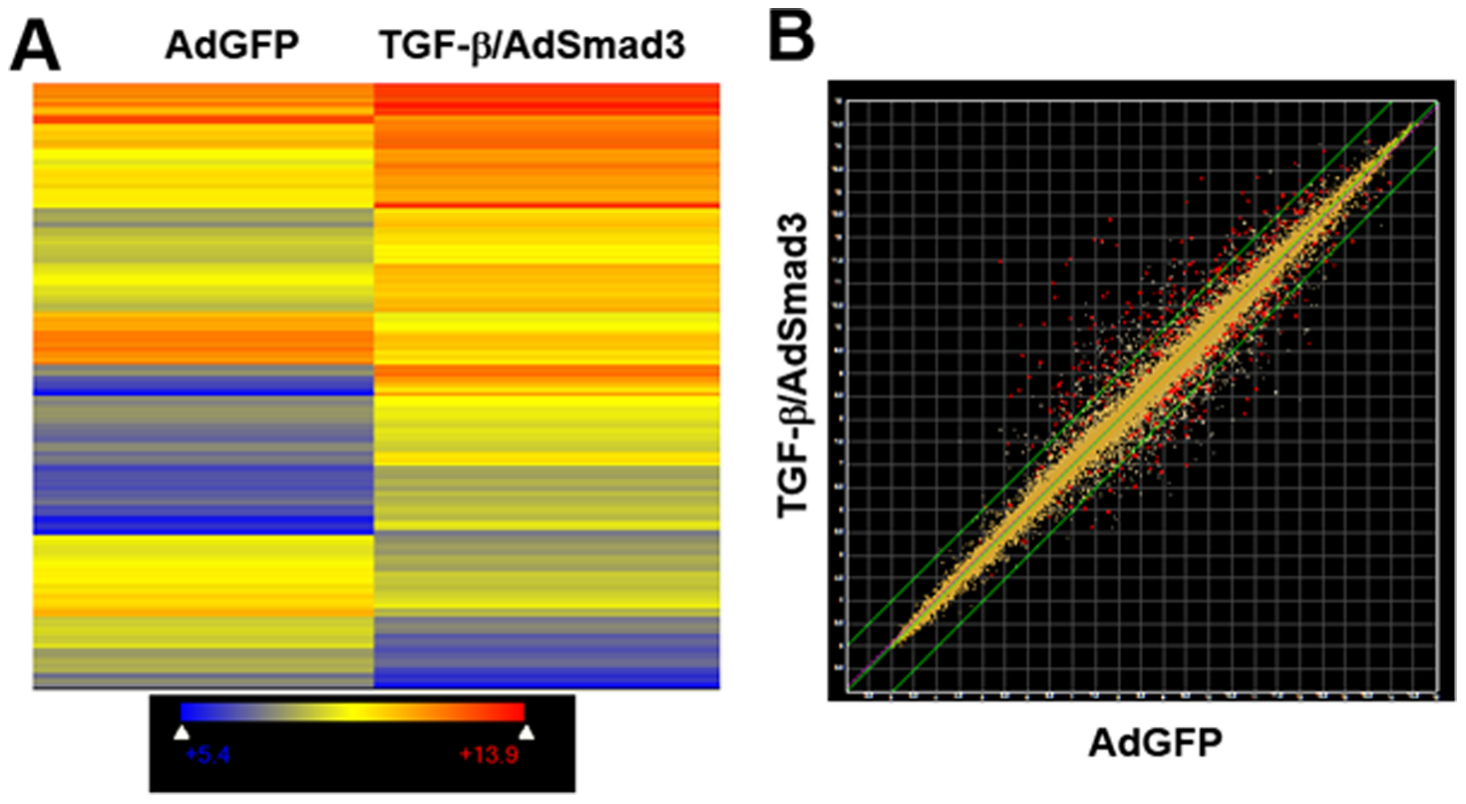
TGF-β/Smad3 treatment significantly affects gene expression in vascular SMCs. Cultured rat vascular SMCs were infected with an adenovirus expressing Smad3 (AdSmad3) and treated with TGF-β (5 ng/ml) for 24 h. Adenovirus expressing GFP (AdGFP) was used as a control. Gene expression was analyzed by microarray (n = 3). **A**. Heat map shows regulated genes that are more than 2-fold and significantly increased or decreased after TGF-β/Smad3 treatment. Color encoded relative gene expression levels are expressed in log2 scale. Blue represents genes with lower expression level whereas red represents genes with higher expression (see legend). **B**. Dot plot overview of up and down regulated genes after TGF-β/AdSmad3 treatment are compared to AdGFP control. Green lines represent unchanged (middle), 2-fold up-regulated (upper-left) or 2-fold down-regulated (lower right) genes. Red dots represent genes that are significantly different from control (P<0.05) and yellow dots represent genes that are not.

The genome-wide changes in mRNA expression that occur in SMCs in response to enhanced TGF-β/Smad3 signaling are of great interest and will provide significant insight into the role of TGF-β in the development of restenosis. To identify which genes are influenced, rat primary SMCs were infected with adenoviral vectors expressing human Smad3 or control GFP. SMC cultures were serum-starved for 24 hours and the Smad3 group was stimulated with TGF-β (5 ng/ml) for 24 hours prior to RNA isolation and Affymetrix gene expression array analysis. By comparing control (AdGFP) SMCs to cells expressing Smad3 and stimulated by TGF-β our goal was to mimic a comparison between uninjured artery with low levels of TGF-β and Smad3 and injured artery with increased expression of both proteins. This combined TGF-β/Smad3 stimulation led to the significant (p<0.05) (>2.0 fold) changes in the expression levels of 219 genes with 143 genes up-regulated and 76 genes down-regulated (Figure 1A). Although we used p<0.05 as our threshold and a two-fold change in expression, 289 additional genes were observed that had a statistically significant (p<0.05) change in gene expression but the numerical change was not greater than 2.0 fold (Figure 1B). Additionally, there were 466 genes that exhibited a greater than 2.0 fold change in gene expression but these differences did not reach statistical significance (Figure 1B). Although the criteria used to select the 219 genes that are the focus of this analysis were reasonably stringent, there are clearly additional genes that are affected by the combination of TGF-β and Smad3. Nevertheless, the findings from this gene expression array demonstrate that combined TGF-β/Smad3 signaling leads to changes in expression of a large number of genes that are potentially responsible for the altered cellular state of SMCs following *in vivo* vascular injury.

### DAVID GO-term Analysis of TGF-β/Smad3 Differentially Regulated Genes

**Figure 2:**
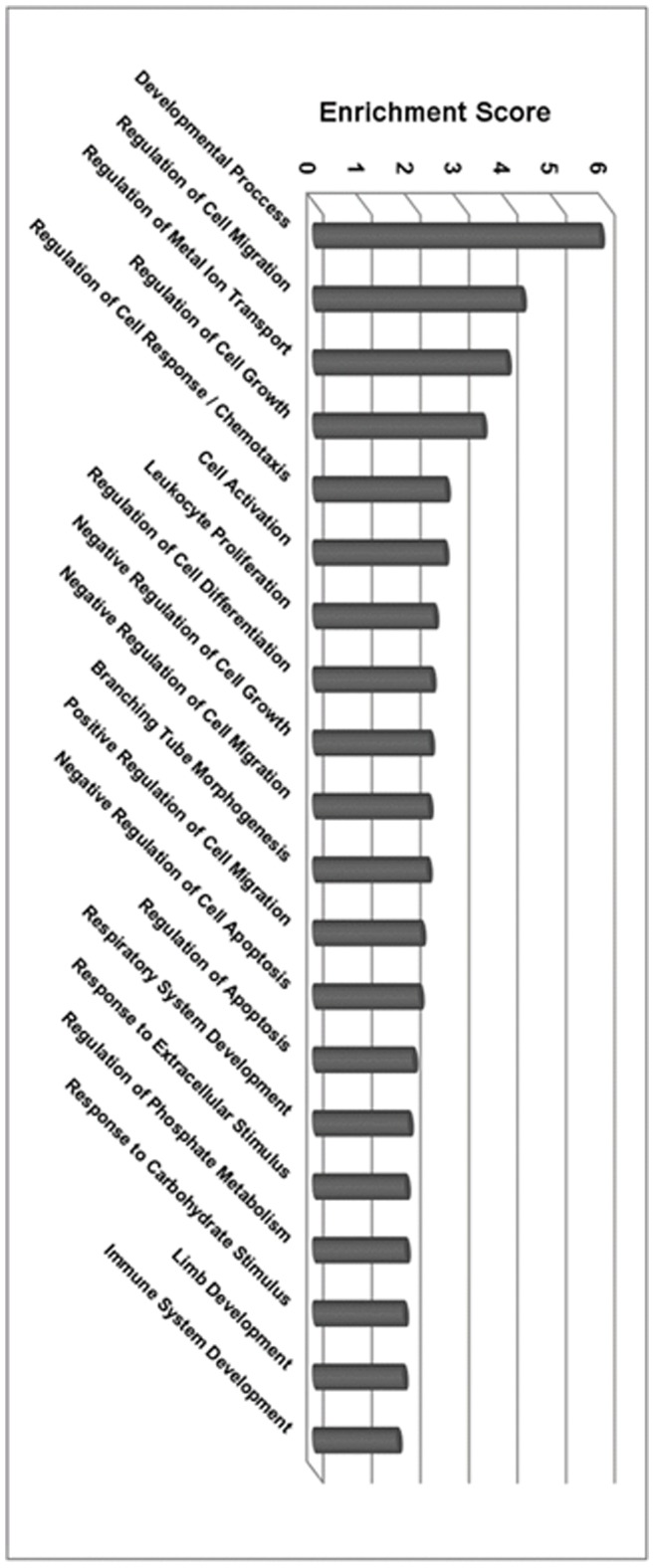
Functional role of TGF-β/Smad3 regulated genes. Significantly regulated genes after AdSmad3/TGF-β treatment (>2-fold change and P<0.05) were submitted to David Bioinformatics Resources 6.7 (NIH, NIAID) for functional analysis. TGF-β/Smad3 regulated genes are associated with multiple functions based upon clustering annotation measured by Fisher Exact in the DAVID system. The enrichment scores represent the relative importance of the function of each the regulated genes. An enrichment score of 1.3 is similar to P<0.05.

Additional studies were then performed to identify the putative biological function of these 219 genes. In order to better understand the consequences of TGF-β/Smad3 activation of SMCs we utilized the DAVID bioinformatics database (http://david.abcc.ncifcrf.gov/) to determine enriched gene ontology (GO) terms. The gene ontology project provides a controlled vocabulary of terms for describing gene product characteristics and gene product annotation data from the published literature. These terms are designed to establish a universal vocabulary for describing the molecular function, biological processes, and cellular component associated with every gene in the genome. For this particular analysis, DAVID identified significantly enriched biological processes associated with our list of 219 differentially expressed genes and clustered these into functional categories. The top 20 functional categories, based on enrichment score, are displayed in Figure 2 and the entire data set can be found in Table S1. Interestingly, the most enriched functional category with an enrichment score of 5.9 is “Developmental Process,” suggesting that SMCs stimulated by TGF-β/Smad3 may reactivate a developmental pattern of gene expression. Included in the top 20 were other functional categories relating to the developmental processes including “Regulation of Cell Differentiation,” “Branching Tube Morphogenesis,” Respiratory System Development,” “Limb Development,” and “Immune system Development” (Figure 2). Although categories related to development of the limb, respiratory, and immune systems seem counterintuitive, it is probable that similar genes function in the development of multiple organ systems, including the vascular system. In sum, these results reinforce the notion that SMCs, when exposed to Smad3 and TGF-β, reactivate a pattern of developmental gene expression.

Following vascular injury and as a precursor to intimal hyperplasia, SMCs migrate, become proliferative and are resistant to apoptosis. In SMCs activated by Smad3 and TGF-β we observed functional categories of genes that promote all three of these processes. Inclusive within the top 20 functional categories were “Regulation of Cell Migration,” “Regulation of Cell Response/Chemotaxis,” “Negative Regulation of Cell Migration” and “Positive Regulation of Cell Migration” showing that multiple genes affected by TGF-β/Smad3 influence SMC migration. Also, “Negative Regulation of Cell Apoptosis” and “Regulation of Apoptosis” indicate that TGF-β/Smad3 may have a significant effect on SMC apoptosis. Finally, “Regulation of Cell Growth” and “Leukocyte Proliferation” suggest that TGF-β/Smad3 activation of SMCs can also influence proliferation. In our previous studies we have demonstrated that TGF-β/Smad3 stimulation is capable of enhancing proliferation and migration and protecting against apoptosis of vascular SMCs thereby contributing to the development of neo-intimal hyperplasia. The identification of these enriched categories provides potential mechanisms for our previous findings.

Taken together, the DAVID GO-term enrichment analysis reinforces that TGF-β/Smad3 activates genes associated with de-differentiation/differentiation as well as SMC proliferation, migration and apoptosis. The sum of these effects may provide an explanation why TGF-β/Smad3 have such a potent stimulatory effect on the development of intimal hyperplasia.

### Categorization of Developmental Related Genes

The “Developmental Process” is the most enriched functional category populated by the 219 differentially expressed genes induced by TGF-β/Smad3. We combined the various functional clusters and generated a list of 60 genes that are related to development/differentiation/or de-differentiation. To better understand the role of these 60 genes in SMC behavior we conducted an extensive literature search and classified each into one of three major categories: “de-differentiation related,” “differentiation related,” or “other developmental related.” TGF-β/Smad3 treatment of SMCs significantly regulated a cluster of 17 genes that have been previously associated with de-differentiation in one or more cell types (Table 1). We also identified an additional 34 genes that, in at least one cellular system, have been found to be associated with cell differentiation (Table 2). There were an additional 9 developmental genes that we could not categorize as associated with de-differentiation or differentiation (Table 3).

**Table 1:** De-differentiation Genes.

Gene Symbol	DAVID Name	Fold Change	p-value
Cxcl12	chemokine (C-X-C motif) ligand 12 (stromal cell-derived factor 1)	−2.79	0.045
Cxcr4	chemokine (C-X-C motif) receptor 4	4.16	0.017
Chrdl2	chordin-like 2	9.77	0.034
Enpp1	ectonucleotide pyrophosphatase/phosphodiesterase 1	2.63	0.045
Fgf1	fibroblast growth factor 1	5.00	0.017
Foxq1	forkhead box Q1	2.25	0.049
Fstl3	follistatin-like 3 (secreted glycoprotein)	3.43	0.042
Grem1	gremlin 1, cysteine knot superfamily, homolog (Xenopus laevis)	−2.33	0.043
Hbegf	heparin-binding EGF-like growth factor	2.80	0.046
Id2	inhibitor of DNA binding 2	−2.25	0.042
Pde3b	phosphodiesterase 3B, cGMP-inhibited	−2.54	0.007
Ptgs2	prostaglandin-endoperoxide synthase 2	2.62	0.043
Pthlh	parathyroid hormone-like hormone	3.02	0.015
Sox18	SRY (sex determining region Y)-box 18	3.19	0.020
Terf1	telomeric repeat binding factor (NIMA-interacting) 1	−2.13	0.045
Wnt2b	wingless-type MMTV integration site family, member 2B	17.02	0.008
Wnt9a	wingless-type MMTV integration site family, member 9A	3.95	0.029

**Table 2:** Differentiation Genes.

Gene Symbol	DAVID Name	Fold Change	p-value
Ada	adenosine deaminase	4.96	0.030
Amtn	Amelotin	68.06	0.012
Ass1	argininosuccinate synthetase 1	23.98	0.013
Bcl2	B-cell CLL/lymphoma 2	−3.18	0.030
Capn5	calpain 5	−2.04	0.042
Cdkn2b	cyclin-dependent kinase inhibitor 2B (p15, inhibits CDK4)	3.21	0.023
Chrnb1	cholinergic receptor, nicotinic, beta 1 (muscle)	2.51	0.029
Col4a1	collagen, type IV, alpha 1	2.11	0.047
Csrp2	cysteine and glycine-rich protein 2	3.10	0.022
Dgkg	diacylglycerol kinase, gamma	−2.05	0.035
Ercc1	excision repair cross-complementing rodent repair deficiency, complementation group 1	2.26	0.021
Fnbp1	formin binding protein 1	−2.20	0.032
Gal	galanin prepropeptide	24.49	0.013
Hells	helicase, lymphoid specific	3.14	0.028
Ifi204	interferon activated gene 204	−2.93	0.024
Lrp8	low density lipoprotein receptor-related protein 8, apolipoprotein e receptor	2.11	0.021
Mdga1	MAM domain containing glycosylphosphatidylinositol anchor 1	3.66	0.012
Meox2	mesenchyme homeobox 2	−3.40	0.034
Mustn1	musculoskeletal, embryonic nuclear protein 1	−3.12	0.019
Myo1e	myosin IE	2.04	0.022
Nfib	nuclear factor I/B	−2.10	0.029
Ngf	nerve growth factor (beta polypeptide)	3.33	0.019
Plaur	plasminogen activator, urokinase receptor	2.23	0.021
Plxna2	plexin A2	3.96	0.016
Plxna4a	plexin A4, A	3.68	0.045
Ppap2b	phosphatidic acid phosphatase type 2B	−5.72	0.022
Prdm1	PR domain containing 1, with ZNF domain	2.47	0.028
Selenbp1	selenium binding protein 1	−6.38	0.035
Sept4	septin 4	−2.26	0.032
Smpd3	sphingomyelin phosphodiesterase 3, neutral	−3.44	0.017
Tgfb1	transforming growth factor, beta 1	2.00	0.025
Unc5c	unc-5 homolog C (C. elegans)	−2.07	0.028
Wnt11	wingless-type MMTV integration site family, member 11	3.82	0.018
Wnt5a	wingless-type MMTV integration site family, member 5A	4.55	0.033
Gene Symbol	DAVID Name	Fold Change	p-value
Ada	adenosine deaminase	4.96	0.030
Amtn	Amelotin	68.06	0.012
Ass1	argininosuccinate synthetase 1	23.98	0.013
Bcl2	B-cell CLL/lymphoma 2	−3.18	0.030
Capn5	calpain 5	−2.04	0.042
Cdkn2b	cyclin-dependent kinase inhibitor 2B (p15, inhibits CDK4)	3.21	0.023
Chrnb1	cholinergic receptor, nicotinic, beta 1 (muscle)	2.51	0.029
Col4a1	collagen, type IV, alpha 1	2.11	0.047
Csrp2	cysteine and glycine-rich protein 2	3.10	0.022
Dgkg	diacylglycerol kinase, gamma	−2.05	0.035
Ercc1	excision repair cross-complementing rodent repair deficiency, complementation group 1	2.26	0.021
Fnbp1	formin binding protein 1	−2.20	0.032
Gal	galanin prepropeptide	24.49	0.013
Hells	helicase, lymphoid specific	3.14	0.028
Ifi204	interferon activated gene 204	−2.93	0.024
Lrp8	low density lipoprotein receptor-related protein 8, apolipoprotein e receptor	2.11	0.021
Mdga1	MAM domain containing glycosylphosphatidylinositol anchor 1	3.66	0.012
Meox2	mesenchyme homeobox 2	−3.40	0.034
Mustn1	musculoskeletal, embryonic nuclear protein 1	−3.12	0.019
Myo1e	myosin IE	2.04	0.022
Nfib	nuclear factor I/B	−2.10	0.029
Ngf	nerve growth factor (beta polypeptide)	3.33	0.019
Plaur	plasminogen activator, urokinase receptor	2.23	0.021
Plxna2	plexin A2	3.96	0.016
Plxna4a	plexin A4, A	3.68	0.045
Ppap2b	phosphatidic acid phosphatase type 2B	−5.72	0.022
Prdm1	PR domain containing 1, with ZNF domain	2.47	0.028
Selenbp1	selenium binding protein 1	−6.38	0.035
Sept4	septin 4	−2.26	0.032
Smpd3	sphingomyelin phosphodiesterase 3, neutral	−3.44	0.017
Tgfb1	transforming growth factor, beta 1	2.00	0.025
Unc5c	unc-5 homolog C (C. elegans)	−2.07	0.028
Wnt11	wingless-type MMTV integration site family, member 11	3.82	0.018
Wnt5a	wingless-type MMTV integration site family, member 5A	4.55	0.033

**Table 3:** Other Developmental Genes.

Gene Symbol	DAVID Name	Fold Change	p-value
Arl4a	ADP-ribosylation factor-like 4A	2.16	0.015
Fras1	Fraser syndrome 1 homolog (human)	−4.32	0.033
Gldn	Gliomedin	11.22	0.030
Lef1	lymphoid enhancer binding factor 1	2.84	0.028
Lyn	v-yes-1 Yamaguchi sarcoma viral related oncogene homolog	2.74	0.021
Pcsk6	proprotein convertase subtilisin/kexin type 6	2.84	0.016
Prkcq	protein kinase C, theta	3.31	0.019
Ptprm	protein tyrosine phosphatase, receptor type, M	2.15	0.029
Slc1a3	solute carrier family 1 (glial high affinity glutamate transporter), member 3	2.50	0.031

For those 17 de-differentiation genes, the degree of de-differentiation might be further subdivided into a hierarchy of progenitor cells being the least de-differentiated, followed by multipotent cells and then pluripotent cells as the most de-differentiated. A single gene was found to be associated with pluripotency. This gene, telomeric repeat binding factor 1 (Terf1), is essential for the protection of telomere ends and induction of induced pluripotent stem cells from somatic precursors [10]. In contrast, we found several genes associated with multipotent hematopoietic stem cells including the stem cell chemoattractant, CXCL12 (SDF-1) and its corresponding receptor CXCR4. Inhibitor of DNA binding 2 (Id2) is also included in the de-differentiation table in that it has been shown to inhibit the differentiation of hematopoietic stem cells into myeloid cells [11]. In addition, several genes associated with multipotent mesenchymal stem cells were also observed. The secreted factors FGF1 and HB-EGF have both been shown to play essential roles in mesenchymal stem cell renewal and inhibition of spontaneous differentiation [12], [13], [14]. Likewise, Gremlin-1 (Grem1) and Wnt9a expression are responsible for maintaining a population of mesenchymal stem cells in a pre-osteoblast state [15], [16] whereas expression of PTH-LH inhibits chondrocyte differentiation from mesenchymal stem cell precursors [17]. We also identified 5 genes from the de-differentiation category that are involved in progenitor cell activity. Interestingly, these genes were shown to inhibit the differentiation of local progenitor cells into their adult counterparts in various tissues including: for skin keratinocytes, Prostaglandin-endoperoxide synthase 2 (Ptgs2); for blood cell progenitors, Sox18; for chondrocyte progenitors, Chordin-like 2 (Chrdl2); and for retinal progenitor cells, Wnt2b [18], [19], [20], [21]. Of most relevance to vascular disease, Forkhead box Q1 (Foxq1) has been shown to inhibit the differentiation of local progenitors into SMCs [22].

Reactivation of development related genes may be of significant consequence after vascular injury. These genes can modulate the de-differentiation of SMCs into stem-like cells which regain plasticity with increased capacity to proliferate and migrate.

### Validation of Array Data for TGF-β/Smad3 Gene Expression by qRT-PCR

**Figure 3:**
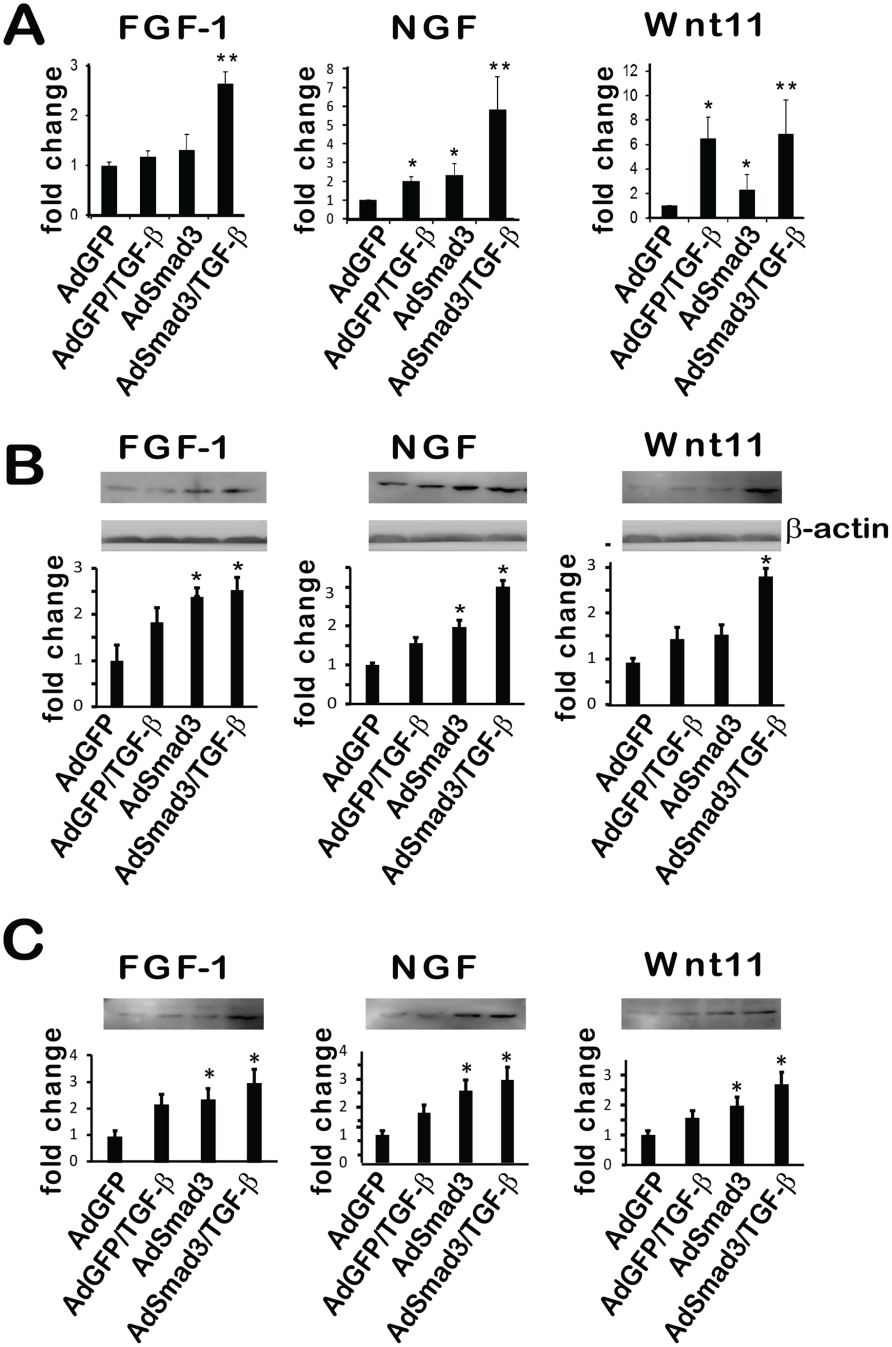
Up-regulation of mRNA and protein levels of FGF-1, NGF and Wnt11 in SMCs by AdSmad3/TGF-β treatment. Rat vascular SMCs were infected with AdSmad3 and treated with TGF-β (5 ng/ml) for 24 h. **A**. qRT-PCR was performed to evaluate gene expression of Fibroblast Growth Factor 1 (FGF1), Nerve Growth Factor (NGF) and wingless-type MMTV integration site family member 11 (Wnt11). **B** and **C**. Western blotting was performed to determine the protein production of these 3 growth factors contained in cell lysates (B) or secreted into media (C). Controls were AdGFP, AdGFP+ TGF-β and AdSmad3. **P<0.05, compared to all controls; *P<.05, compared to AdGFP; n = 3.

Quantitative RT-PCR (qRT-PCR) was performed to corroborate our microarray findings for TGF-β/Smad3 regulated gene expression. The Affymetrix gene array revealed a number of intriguing genes regulated by TGF-β/Smad3 and up-regulation of several that have been previously reported to be associated with de-differentiation. To confirm the microarray results, SMCs were infected with AdGFP or AdSmad3 and then treated with solvent or TGF-β. The mRNA levels of several highly regulated genes associated with development were evaluated by qRT-PCR (Figure 3). We initially evaluated three genes due to their well-established roles in the development of a variety of mammalian tissue including vascular, epithelium, and neuronal tissue. The results of qRT-PCR were in agreement with microarray results with regard to the expression of fibroblast growth factor 1 (FGF1), nerve growth factor (NGF) and wingless-related MMTV integration site 11 (Wnt11). FGF1 was elevated ∼2.5 fold in TGF-β/Smad3-stimulated cells compared to GFP control, whereas NGF and Wnt11 were up-regulated ∼6 fold and ∼7 fold, respectively (Figure 3). Although all these genes were elevated, there were differences in the expression profiles of each. For Wnt11, TGF-β alone produced an effect on gene expression that was almost as significant as that found with combined TGF-β/Smad3 stimulation. In contrast, FGF1 and NGf up-regulation by TGF-β was heavily reliant on the presence of elevated levels of Smad3 (Figure 3A). We further confirmed up-regulation of protein production of these 3 factors. Western blotting showed an ∼2.5–3 fold increase of all 3 proteins either in SMC lysates (Figure 3B) or cell culture media (Figure 3C), as a result of TGF-β/Smad3 treatment. Following the treatment with AdSmad3 alone (no TGF-β), while only FGF-1 was significantly up-regulated in cell lysates, all 3 factors were significantly increased in conditioned media.

**Figure 4:**
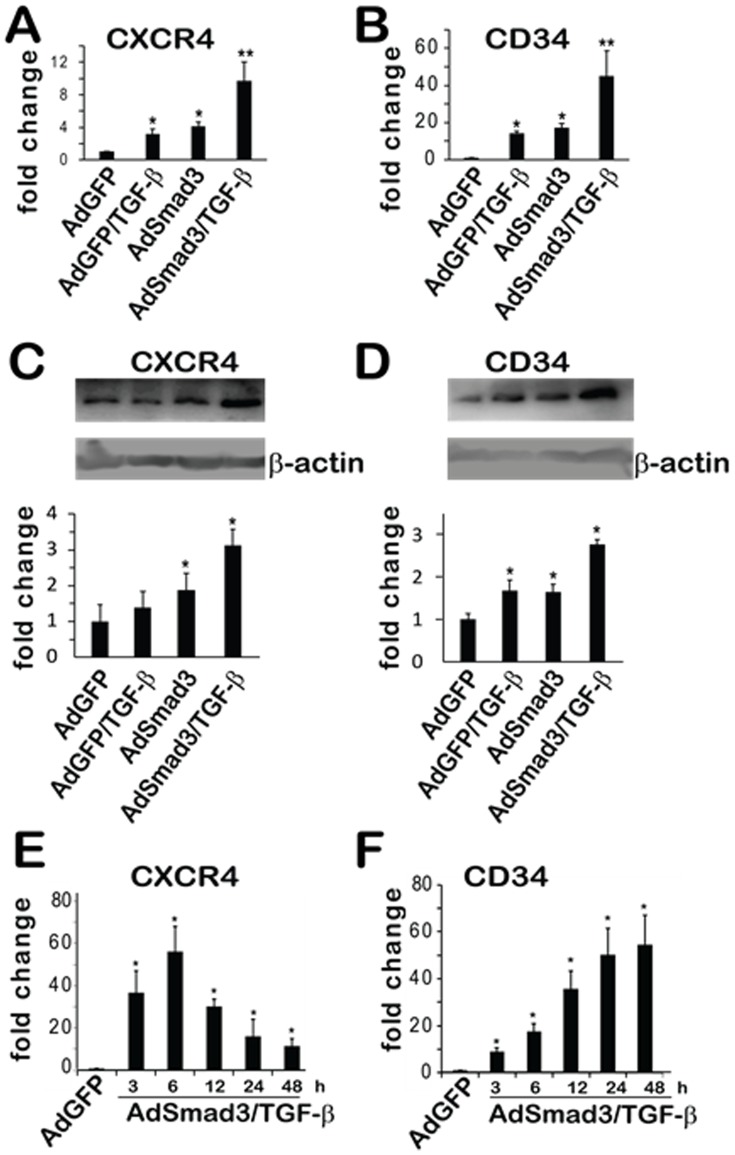
Up-regulation of mRNA and protein levels of CD34 and CXCR4 in SMCs by AdSmad3/TGF-β treatment. Rat vascular SMCs were infected with AdSmad3 and treated with TGF-β (5 ng/ml) for 24 h. **A**. qRT-PCR was performed to evaluate gene expression of CD34 and CXCR4. **B**. Western blotting was performed to determine the protein production of CD34 and CXCR4 in cell lysates. **C**. Time course of CD34 and CXCR4 gene expression after TGF-β treatment of AdSmad3-infected SMCs. Controls were AdGFP, AdGFP+ TGF-β and AdSmad3. **P<0.05, compared to all controls; *P<.05, compared to AdGFP; n = 3.

In additional experiments we evaluated expression of CXCR4 and CD34, both stem/progenitor associated genes. The qRT-PCR data indicate that CXCR4 and CD34 mRNA levels were increased by ∼10 fold and 45 fold, respectively, after TGF-β/Smad3 stimulation (Figure 4A); Western blotting confirmed an increase of these two proteins in cell lysates albeit to a lower extent (∼3 fold) (Figure 4B). In order to understand the temporal expression profiles of these two genes, we determined the qRT-PCR time course. SMCs were infected with AdSmad3 and treated with TGF-β (5ng/ml) for the indicated times, AdGFP served as a control. TGF-β/Smad3 gradually and consistently increased CD34 expression to 30 fold at 12 hours and to a maximum of 50 fold at 48 hours. In contrast, CXCR4 was enhanced and peaked at 50 fold at 6 hours then gradually fell to 10-fold by 48 hours (Figure 4C). These results further validate the array findings and confirm that TGF-β/Smad3 activate two additional genes associated with cell development. Interestingly, the time course of each differs with CXCR4 associated with a peak in expression at 6 hours and CD34 expression gradually increasing over time.

**Figure 5:**
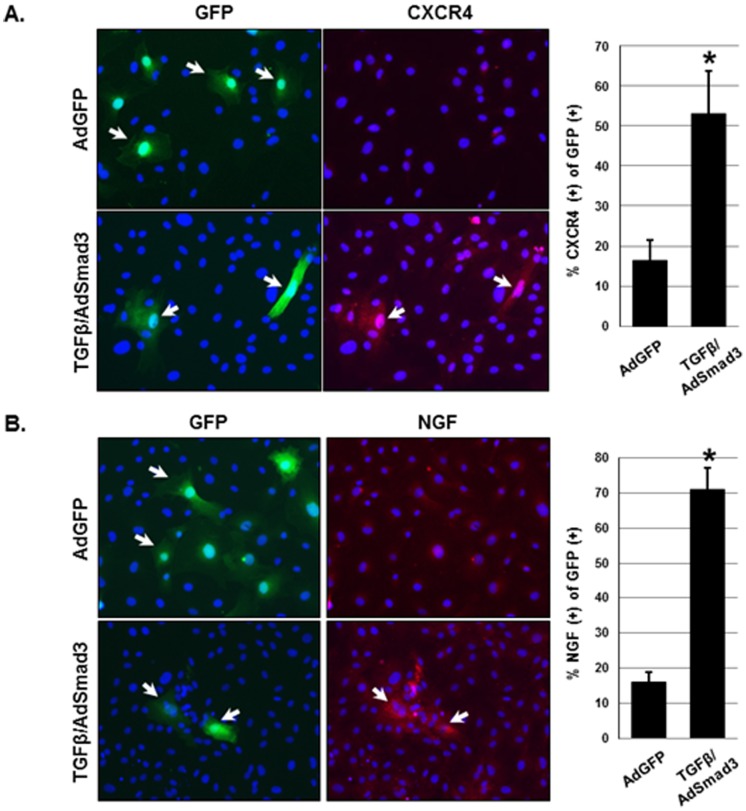
Immunocytochemistry detection of CXCR4 and NGF up-regulated by AdSmad3/TGF-β treatment. Rat vascular SMCs were infected with AdGFP or AdSmad3 and treated with TGF-β (5 ng/ml) for 24 hrs. Cells were fixed and subjected to immunostaining for CXCR4 (**A**) or NGF (**B**) as described in detail in Materials and Methods. CXCR4 or NGF positive cells were quantified as percent of GFP fluorescent cells. *P<0.05 compared to AdGFP, n = 3.

Using immunocytochemistry we then determined the percentage of SMCs that express development/progenitor associated genes in response to the combined stimulation of AdSmad3 and TGF-β. In order to avoid an interference of GFP fluorescence with microscopic detection of immunostained proteins, we sought to use GFP as a surrogate of viral expression of Smad3 because the adenoviral vector was designed to co-express GFP and Smad3. Indeed, after AdSmad3 infection and TGF-β treatment, over 90% of GFP-expressing SMCs were Smad3-overexpressing cells (Figure S1 in File S1). We then immunostained two representative factors, NGF and CXCR4, in AdGFP and AdSmad3 infected (and TGF-β treated) cells. We found that ∼60% of GFP cells were NGf and CXCR4 positive cells (Figure 5), indicating a strong association of up-regulation of these factors with Smad3 expression.

In sum, through determination of mRNA, protein, and *in situ* cellular expression, we have confirmed significant up-regulation of five genes, all of which were found by the Affymetrix gene expression array to be associated with SMC development.

### TGF-β/Smad3 Negatively Regulates SMC Differentiation

**Figure 6:**
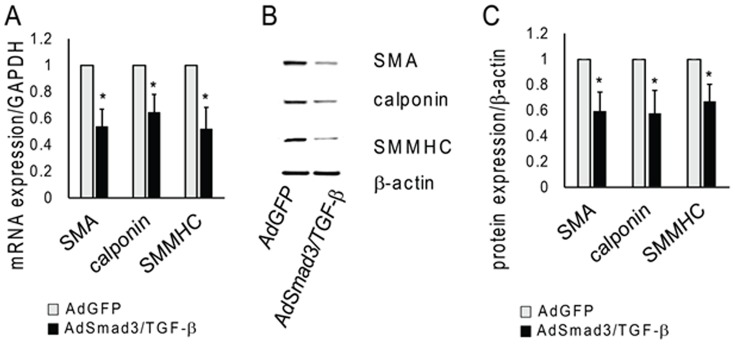
TGF-β/Smad3 treatment affects expression of SMC contractile proteins. Rat vascular SMCs were infected with AdSmad3 and treated with TGF-β (5 ng/ml) for 24 hrs. **A**. qRT-PCR expression of smooth muscle actin (SMA), calponin, and smooth muscle myosin heavy chain (SMMHC) (n = 3; *P<0.05 compared to AdGFP). **B**. Representative examples of Western blotting of SMA, calponin and SMMHC (n = 3). **C**. Quantification of Western Blotting (*P<0.05 compared to AdGFP).

TGF-β is classically considered a stimulant of SMC differentiation [23], [24], [25], [26]. However, the foregoing suggests that in the context of elevated Smad3, TGF-β promotes de-differentiation. Consistent with this hypothesis, we propose that TGF-β/Smad3 might also inhibit SMC differentiation. To explore this possibility, using qRT-PCR we evaluated the effect of TGF-β/Smad3 on expression of smooth muscle actin (SMA), Calponin, and smooth muscle myosin heavy chain (SM-MHC), which are three proteins known to be associated with SMC differentiation. SMCs were infected with AdSmad3 and treated with TGF-β (5 ng/ml) for 24 h or infected with AdGFP. qRT-PCR revealed that activation of the TGF-β/Smad3 pathway significantly decreased the expression of the SMC contractile genes, SMA, Calponin, SM-MHC to approximately 60% of control (Figure 6A); either AdSmad3 or TGF-β alone did not produce a significant change (Figure S2 in File S1). These findings were then confirmed by Western blotting, using an identical experimental protocol. Protein levels associated with each of these genes fell to approximately 50% of control (Figure 6B, C). Thus, our findings suggest that TGF-β/Smad3 not only enhance expression of factors that promote cellular de-differentiation but also inhibit factors that have been previously found to be associated with differentiation or preservation of the SMCs in a quiescent, contractile state. Whereas TGF-β alone has been strongly associated with SMC differentiation, the sum of our findings suggest that in the presence of elevated Smad3, TGF-β transforms into a potent stimulant of SMC de-differentiation.

## Discussion

TGF-β is a critically important factor in the development of intimal hyperplasia. Its expression is up-regulated in arteries and veins following vascular reconstruction. Moreover, increasing TGF-β through viral expression in an animal model of arterial injury exacerbates intimal hyperplasia, whereas inhibiting TGF-β signaling by using antibodies or by expressing a soluble form of the TGF-β receptor attenuates it [8]. These findings are surprising since, TGF-β, *in vitro,* is a potent inhibitor of SMC proliferation and migration, both necessary contributors to intimal hyperplasia. Our recent published findings bring some resolution to this conundrum. It appears that TGF-β in the presence of elevated levels of its canonical signaling protein Smad3, transforms from an inhibitor of SMC proliferation and migration to a stimulant of both processes. We have previously demonstrated that administration of TGF-β to cultured SMCs coupled with adenoviral overexpression of Smad3, leads to enhanced proliferation, migration, and synthesis of extracellular matrix [6], [27], [28], [29]. These results are mirrored *in vivo* where overexpression of Smad3 was found to enhance balloon injury-induced intimal hyperplasia in rat carotid arteries [6], [29]. While transfection with siRNA to reduce Smad3 levels reversed SMC proliferation stimulated by AdSmad3 [63], adenoviral expression of Smad7, which blocks Smad3 signaling, effectively inhibited intimal hyperplasia [6].

Despite the current knowledge that TGF-β/Smad3 play crucial roles in stimulating SMC proliferation, migration and intimal hyperplasia in response to arterial injury, changes in gene expression and the mechanism behind this response remain elusive. We performed the Affymetrix gene expression arrays on SMCs infected with AdSmad3 and stimulated by TGF-β, with the assumption that identification of genes regulated by the combination of TGF-β/Smad3 may provide important insight into how this signaling axis affects intimal hyperplasia. We identified 219 genes whose expression was substantially altered (>2 fold change, p<0.05). We then applied DAVID bioinformatics tools to analyze TGF-β/Samd3 regulated gene functions. The most salient finding of this analysis was that the most prominently regulated genes were those related to development, some of which are associated with stem or progenitor cells. It is likely that expression of these developmental and stem cell-related genes is indicative that TGF-β/Smad3 at minimum produces SMC de-differentiation or at maximum transforms SMCs into stem or progenitor cells following vascular injury.

It has been thought for many years that for neointimal hyperplasia to occur, a transformation of medial SMCs is necessary to allow for their migration to and proliferation within the subintimal space. Classically, this process has been described as “phenotypic switching” or “phenotypic modulation” whereby SMCs transition from quiescent and contractile to synthetic cells following vascular injury [2], [30]. As a consequence, SMCs reduce their expression of contractile genes, most notably smooth muscle alpha-actin, calponin, and smooth muscle myosin heavy chain while up-regulating expression of genes that lead to the production of extracellular matrix [2], [30]. A variety of factors released at the time of arterial injury, including PDGF-BB, PDGF-DD, oxidized phospholipids and extracellular matrix proteins, have been implicated in the transformation of SMCs from a contractile to a synthetic phenotype [31], [32], [33], [34]. Furthermore, many of these stimulants exert their function through the intra-cellular signaling pathways, ERK, p38 MAPK, and Akt, producing their profound effects on cellular gene expression, proliferation, and migration [32], [35], [36].

Our data show that TGF-β/Smad3 in SMCs can re-activate the expression of development/stem cell related genes which may in part account for the SMC phenotype transition to a more de-differentiated state. The concept that SMCs express stem cell-related genes following arterial injury is not entirely novel and has been suggested by recent studies. Our group has previously reported the presence of the stem cell factor, C-kit, in vascular SMCs derived from human saphenous vein. Moreover, we found a substantial increase in C-kit in hyperplastic plaque that develops in the rat carotid model of intimal hyperplasia [37]. Ferlosio *et al.* demonstrated an increase in the percentage of medial cells expressing the stem cell markers flt-1 and c-kit in large arteries of aged humans and rats versus younger controls [38]. SMCs isolated from aged rats were also more proliferative, migratory and expressed less SMC alpha actin compared to younger controls [38]. These findings reinforce the notion that SMCs up-regulate stem cell-associated genes in response to injury or chronic stress such as aging.

Additional evidence that SMCs can activate stem cell gene expression following arterial injury is revealed through numerous studies of the epigenetic regulator, Klf4. Several publications by the Owens group have demonstrated that Klf4 is up-regulated by PDGF-BB and plays a pivotal role in phenotypic transformations of SMCs [39], [40]. These studies are especially interesting in that Klf4 is one of the four “Yamanaka factors” that are capable of re-programming somatic cells into induced pluripotent stem cells. The role of Klf4 in SMC de-differentiation, however, is still somewhat unclear. Other studies have shown that Klf4 has an inhibitory effect on SMC proliferation [41]. The discrepant role of Klf4 in SMC differentiation/de-differentiation is discussed in a recent review where it is suggested that post-translational modifications of this protein might explain its variable function [42]. Regardless, the stem cell marker Klf4 appears to play an important, but somewhat controversial, role in SMC differentiation, proliferation, and vascular restenosis.

PDGF-BB is by far the most widely studied stimulant of de-differentiation and numerous investigators have dissected the molecular mechanisms through which this protein induces phenotypic switching of SMCs. PDGF-BB has been shown to activate the MAPK signaling cascade leading to Elk-1 phosphorylation [43]. Myocardin is a master regulator for differentiation and contractile protein expression in SMCs [46]. Phosphorylated Elk-1 displaces myocardin at the promoter of SMC contractile genes and reduces its expression [44]. IL-1β has also been shown in numerous studies to push cells towards a de-differentiated state by inhibiting myocardin. This effect is mediated through NF-kB and Notch3 [45]. These findings demonstrate that there is significant cross-talk between signaling proteins, suggesting that SMC phenotypic switching is a highly dynamic process that involves multiple pathways.

The role of TGF-β in SMC differentiation is controversial and appears to depend upon the cell-type and developmental stage. *In vitro*, TGF-β has been found to stimulate SMC differentiation as evidenced by enhanced contractile protein expression. Chen *et al.* showed that TGF-β signaling promotes contractile protein expression in neural crest (Monc-1) cells which leads to their differentiation into SMCs [24]. Imamura *et al.* showed that TGF-β has the capacity to induce differentiation of bone marrow derived progenitor cells toward a SMC lineage through myocardin. Alternatively, our data suggest that TGF-β and Smad3 promote de-differentiation of adult SMCs, at least partially by reactivating expression of genes related to embryonic development and suppressing expression of SMC differentiation markers. Thus in the presence of elevated Smad3, TGF-β switches from a stimulant of differentiation to a promoter of de-differentiation.

It has been previously discovered that in some cells, TGF-β positively regulates stem cell function. For example, TGF-β signaling is necessary for proliferation of mesenchymal stem cells [47]. Moreover, the TGF-β family has been implicated in the maintenance of embryonic stem cells [48], preventing these cells from differentiating and migrating. Falk *et al.* reported that TGF-β regulates neuronal stem cell proliferation through Wnt signaling [49]. Coincidentally, we found that TGF-β/Smad3 stimulate Wnt, Sox, and FGF family gene expression (Table 2). It appears that elevated Smad3 is crucial for TGF-β regulated de-differentiation. Phosphorylation and nuclear translocation of Smad3 have been observed in stem cells and both are decreased when stem cells differentiate [50], suggesting that activated TGF-β signaling is required to maintain de-differentiation. TGF-β regulates embryonic stem cell function by clustering Smad3 with other key regulators including Nanog and Oct4 [51], [52]. Together, Nanog and Oct4, along with Lin28 and Sox2, can induce differentiated cells into pluripotent stem cells with full capacity to differentiate into any cell type [53].

Our findings demonstrate that reactivation of embryonic developmental genes by TGF-β/Smad3 in a similar manner, leads to SMC plasticity and its consequences including SMC proliferation. Our qRT-PCR data confirm the microarray results and support a positive role for TGF-β/Smad3 in regulating expression of stem cell-related genes and SMC de-differentiation. It is interesting to note that CXCR4 and CD34, which are stem cell/progenitor markers, are substantially up-regulated in SMCs following treatment with TGF-β/Smad3. Furthermore, we found that FGF-1, a potent mitogen for hematopoietic stem cell expansion, was significantly up-regulated following TGF-β/Smad3 stimulation. In contrast to FGF-1, basic fibroblast growth factor (FGF-2) is widely studied in SMCs and is known to enhance proliferation, migration, and drive intimal hyperplasia. Although there is a paucity of research regarding FGF-1 in SMCs, a report by Takahashi *et al.* demonstrated that FGF-1 expression was sufficient to inhibit the differentiation of SMCs from progenitor-like cells [54]. CD34 and CXCR4 have also been studied in the vascular system. CD34 is a well-known marker for hematopoietic stem cells and is often found in endothelial cells [55]. However, our finding of CD34 in SMCs is novel. Several reports have shown that the SDF-1a/CXCR4 axis is important in the development of intimal hyperplasia [56]. Nevertheless, the finding of CXCR4 in SMCs is also relatively novel. In contrast to stem/progenitor markers, SMA, calponin and MHC, signature markers of SMC differentiation, are all down-regulated in SMCs in response to the treatment with TGF-β/Smad3. A decrease of these SMC markers has been commonly used as an indicator of SMC de-differentiation and is often observed in proliferating and migrating SMCs. The sum of our data reveals down-regulation of markers of SMC differentiation and up-regulation of stem/progenitor markers that are not typically expressed in mature SMCs. Thus, it is reasonable to postulate that TGF-β in the presence of elevated Smad3 triggers a process whereby SMCs de-differentiate toward an earlier developmental stage.

In addition to the foregoing factors we also observed increased expression of NGF and Wnt11. While the functions of these two genes in SMCs are not currently known, they have been reported to play a role in differentiation of non-SMC cell types [57], [58], not supporting the hypothesis that TGF-β/Smad3 promotes de-differentiation. However, many developmental genes play differing or even opposite roles depending upon the context or the cell type. It may well be that NGF and Wnt11 in SMCs promote de-differentiation. Alternatively, it might be that TGF-β/Smad3 induce transcription of genes associated with differentiation (NGF and Wnt11) as well as de-differentiation, but the balance of their combined effect favors de-differentiation. It is also interesting to note that TGF-β/Smad3 significantly increased (by ∼5–10 fold compared to AdGFP control) the expression of osteopontin, nestin, and collagen type II, which are markers of osteoblasts, neurocrest cells, and chondrocytes, respectively (Figure S3 in File S1). Moreover, TGF-β/Smad3-conditioned media were able to up-regulate osteopontin and nestin as well (Figure S4 in File S1). Up-regulation of these genes of different lineages lends additional evidence to our assessment that TGF-β/Smad3-stimulated cells were transformed to a progenitor-like state. It is reasonable to postulate that de-differentiated cells have a greater potential than differentiated SMCs to express marker genes of other cell types. Taken together, the foregoing results prompt us to propose that enhanced TGF-β/Smad3 signaling confers on SMCs the propensity to de-differentiate toward an earlier stage in the developmental lineage, which is consistent with our earlier observations that TGF-β/Smad3 promotes SMC proliferation and migration and intimal hyperplasia.

Our primary hypothesis is that SMCs that contribute to intimal hyperplasia are initially differentiated but transform into a de-differentiated state in response to factors such as TGF-β/Smad3. Alternative hypotheses have been proposed that might explain the presence of de-differentiated cells in arterial media and subintimal plaque. One possibility is that these de-differentiated cells translocate from another site. For example, there is substantial evidence that at least some cells that contribute to the neointima are myofibroblasts derived from the arterial adventitia [59]. There are a series of studies showing in the murine model, that neo-intimal cells are, in part, derived from the bone marrow [7], [60], [61], [62]. A recent study raised the controversial notion that de-differentiation of mature SMCs is not the primary driving force behind the development of intimal hyperplasia [3]. Rather, neointima develops from undifferentiated multipotent vascular stem cells (MVSCs) that naturally reside in the media of the normal uninjured artery and then become activated with vascular injury [3]. Although this notion is highly contentious, our findings are not at complete odds with this theory. In studies of animal as well as human plaque, we have found that some but far from all cells, have enhanced expression of Smad3 [63]. Possibly up-regulation of Smad3 signaling is confined to MVSCs following vascular injury. Although the classical concept that quiescent medial SMCs transform into the cells that comprise subintimal plaque is still well accepted, the contribution of de-differentiated cells derived from other sources including the adventitia, bone marrow, or an intrinsic population of stem cells within the normal arterial wall requires further exploration.

TGF-β/Smad3 play an important role in the development of vascular restenosis following arterial reconstruction. Our findings suggest an important role for TGF-β/Smad3 in de-differentiation of vascular SMCs placing the TGF-β/Smad3 signaling axis at the center of a novel pathway that drives a phenotypic switch of vascular SMCs following arterial injury. We have identified a group of novel TGF-β/Smad3 regulated genes that may be central to this process. Blocking de-differentiation is a strategy that might be used to prevent the development of intimal hyperplasia. Thus, a thorough understanding of the de-differentiation factors that are enhanced or the differentiation factors that are suppressed could lead to novel therapeutics to prevent restenotic disease. An approach that includes simultaneous suppression or accentuation of several of these factors might be particularly strategic. The identification of global targets and molecular pathways associated with TGF-β/Smad3 pathway will provide a better understanding of the multi-faceted roles of TGF-β in complex pathophysiological processes of restenosis.

## Supporting Information

Table S1 **The entire data set of microarray.**
(XLSX)

File S1 **Supporting Information Figures. Figure S1, Immunocytochemistry to detect Smad3 overexpression in AdSmad3-infected SMCs.** Rat vascular SMCs were infected with AdGFP or AdSmad3 and treated with TGF-β (5 ng/ml) for 24 h. Cells were fixed and subjected to immunostaining for Smad3 as described in detail in Materials and Methods. Smad3 positive cells were quantified as percent of GFP fluorescent cells. *P<0.05 compared to AdGFP, n = 3. **Figure S2, Evaluation of gene expression of SMC markers regulated by AdSmad3/TGF-β treatment.** Rat vascular SMCs were infected with AdSmad3 and treated with TGF-β (5 ng/ml) for 24 h (red). Controls were AdGFP (light green), AdGFP+ TGF-β (dark green) and AdSmad3 (pink). qRT-PCR was performed to evaluate gene expression of three SMC markers. *P<.05, compared to AdGFP; n = 3. **Figure S3, AdSmad3/TGF-β treatment stimulates expression of chondrocyte, neurocrest, and osteopontin lineage markers.** Rat vascular SMCs were infected with AdSmad3 and treated with TGF-β (5 ng/ml) for 24 h. qRT-PCR was performed to evaluate gene expression of collagen type II, nestin, and osteopontin, which are established markers for chodrocytes, neurocrest cells, and osteoblasts, respectively. Each bar represents a mean ± SD (n = 3). *P<0.05, compared to AdGFP control. **Figure S4, AdSmad3/TGF-β-conditioned media stimulates expression of osteopontin and nestin in naïve SMCs.** Rat vascular SMCs were infected with AdSmad3 and treated with TGF-β (5 ng/ml) for 48 h. Controls were AdGFP, AdGFP+ TGF-β and AdSmad3. Conditioned media collected from those cultures were added to naïve SMCs and incubated for 24 h. qRT-PCR was then performed to evaluate gene expression of osteopontin and nestin. *P<.05, compared to AdGFP; n = 4.
(DOCX)
