# Correction: TGF-β/Smad3 Stimulates Stem Cell/Developmental Gene Expression and Vascular Smooth Muscle Cell De-Differentiation

**DOI:** 10.1371/journal.pone.0103796

**Published:** 2014-07-22

**Authors:** The PLOS ONE Staff

There are errors in Table 2 and Figure 3. In Table 2, the contents are incorrectly duplicated. In Figure 3C, the western blot representing FGF-1 is a duplicate of the one correctly representing Wnt11 in Figure 3B.

Please see the correct Table 2 here.

**Table 2:** Differentiation Genes.

Gene Symbol	DAVID Name	Fold Change	p-value
Ada	adenosine deaminase	4.96	0.030
Amtn	amelotin	68.06	0.012
Ass1	argininosuccinate synthetase 1	23.98	0.013
Bcl2	B-cell CLL/lymphoma 2	−3.18	0.030
Capn5	calpain 5	−2.04	0.042
Cdkn2b	cyclin-dependent kinase inhibitor 2B (p15, inhibits CDK4)	3.21	0.023
Chrnb1	cholinergic receptor, nicotinic, beta 1 (muscle)	2.51	0.029
Col4a1	collagen, type IV, alpha 1	2.11	0.047
Csrp2	cysteine and glycine-rich protein 2	3.10	0.022
Dgkg	diacylglycerol kinase, gamma	−2.05	0.035
Ercc1	excision repair cross-complementing rodent repair deficiency, complementation group 1	2.26	0.021
Fnbp1	formin binding protein 1	−2.20	0.032
Gal	galanin prepropeptide	24.49	0.013
Hells	helicase, lymphoid specific	3.14	0.028
Ifi204	interferon activated gene 204	−2.93	0.024
Lrp8	low density lipoprotein receptor-related protein 8, apolipoprotein e receptor	2.11	0.021
Mdga1	MAM domain containing glycosylphosphatidylinositol anchor 1	3.66	0.012
Meox2	mesenchyme homeobox 2	−3.40	0.034
Mustn1	musculoskeletal, embryonic nuclear protein 1	−3.12	0.019
Myo1e	myosin IE	2.04	0.022
Nfib	nuclear factor I/B	−2.10	0.029
Ngf	nerve growth factor (beta polypeptide)	3.33	0.019
Plaur	plasminogen activator, urokinase receptor	2.23	0.021
Plxna2	plexin A2	3.96	0.016
Plxna4a	plexin A4, A	3.68	0.045
Ppap2b	phosphatidic acid phosphatase type 2B	−5.72	0.022
Prdm1	PR domain containing 1, with ZNF domain	2.47	0.028
Selenbp1	selenium binding protein 1	−6.38	0.035
Sept4	septin 4	−2.26	0.032
Smpd3	sphingomyelin phosphodiesterase 3, neutral	−3.44	0.017
Tgfb1	transforming growth factor, beta 1	2.00	0.025
Unc5c	unc-5 homolog C (C. elegans)	−2.07	0.028
Wnt11	wingless-type MMTV integration site family, member 11	3.82	0.018
Wnt5a	wingless-type MMTV integration site family, member 5A	4.55	0.033

Please see the correct Figure 3 here.

**Figure 3:**
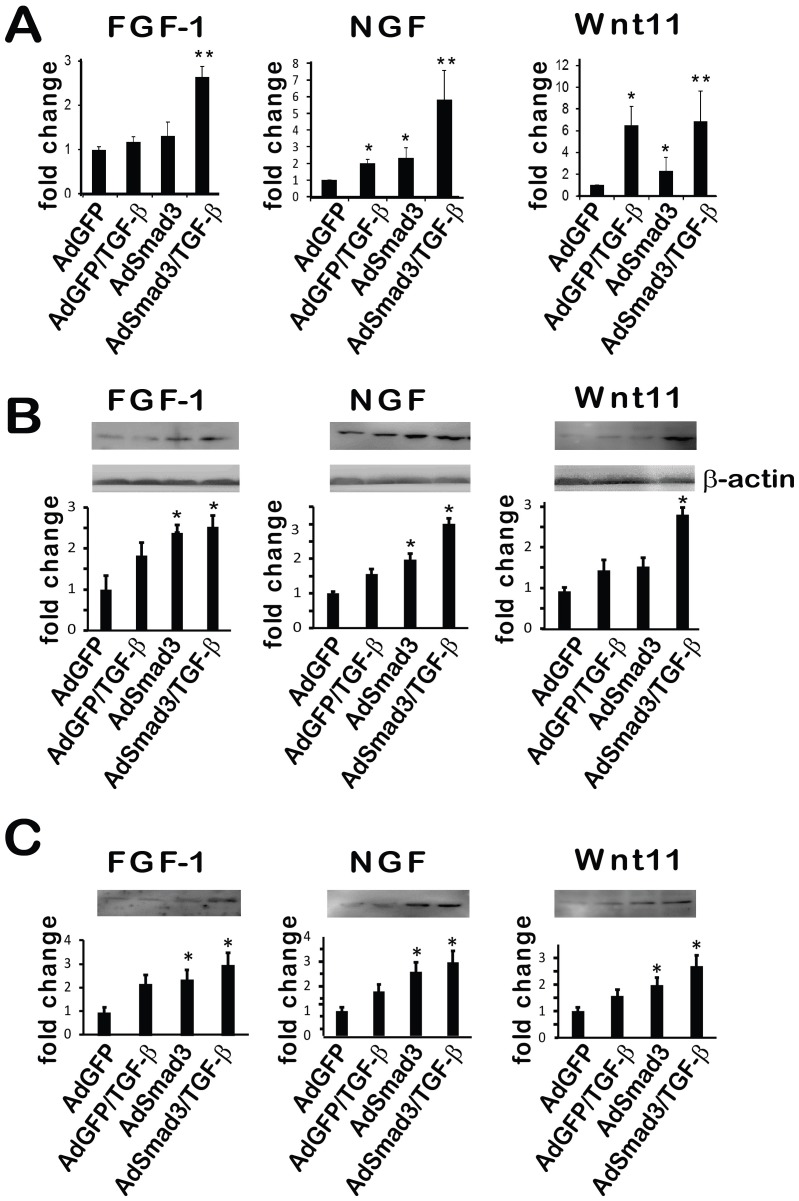
Up-regulation of mRNA and protein levels of FGF-1, NGF and Wnt11 in SMCs by AdSmad3/TGF-β treatment. Rat vascular SMCs were infected with AdSmad3 and treated with TGF-β (5 ng/ml) for 24 h. **A**. qRT-PCR was performed to evaluate gene expression of Fibroblast Growth Factor 1 (FGF1), Nerve Growth Factor (NGF) and wingless-type MMTV integration site family member 11 (Wnt11). **B** and **C**. Western blotting was performed to determine the protein production of these 3 growth factors contained in cell lysates (B) or secreted into media (C). Controls were AdGFP, AdGFP+ TGF-β and AdSmad3. **P<0.05, compared to all controls; *P<.05, compared to AdGFP; n  =  3.
