# Supplementary material for: TGF-β/Smad3 Stimulates Stem Cell/Developmental Gene Expression and Vascular Smooth Muscle Cell De-Differentiation
Source: PLoS One. 2014 Apr 9;9(4):e93995. doi: 10.1371/journal.pone.0093995 (PMC3981734; doi:10.1371/journal.pone.0093995)
Supplement: File S1 — Supporting Information Figures. Figure S1, Immunocytochemistry to detect Smad3 overexpression in AdSmad3-infected SMCs. Rat vascular SMCs were infected with AdGFP or AdSmad3 and treated with TGF-β (5 ng/ml) for 24 h. Cells were fixed and subjected to immunostaining for Smad3 as described in detail in Materials and Methods. Smad3 positive cells were quantified as percent of GFP fluorescent cells. *P<0.05 compared to AdGFP, n = 3. Figure S2, Evaluation of gene expression of SMC markers regulated by AdSmad3/TGF-β treatment. Rat vascular SMCs were infected with AdSmad3 and treated with TGF-β (5 ng/ml) for 24 h (red). Controls were AdGFP (light green), AdGFP+ TGF-β (dark green) and AdSmad3 (pink). qRT-PCR was performed to evaluate gene expression of three SMC markers. *P<.05, compared to AdGFP; n = 3. Figure S3, AdSmad3/TGF-β treatment stimulates expression of chondrocyte, neurocrest, and osteopontin lineage markers. Rat vascular SMCs were infected with AdSmad3 and treated with TGF-β (5 ng/ml) for 24 h. qRT-PCR was performed to evaluate gene expression of collagen type II, nestin, and osteopontin, which are established markers for chodrocytes, neurocrest cells, and osteoblasts, respectively. Each bar represents a mean ± SD (n = 3). *P<0.05, compared to AdGFP control. Figure S4, AdSmad3/TGF-β-conditioned media stimulates expression of osteopontin and nestin in naïve SMCs. Rat vascular SMCs were infected with AdSmad3 and treated with TGF-β (5 ng/ml) for 48 h. Controls were AdGFP, AdGFP+ TGF-β and AdSmad3. Conditioned media collected from those cultures were added to naïve SMCs and incubated for 24 h. qRT-PCR was then performed to evaluate gene expression of osteopontin and nestin. *P<.05, compared to AdGFP; n = 4. (DOCX) [file pone.0093995.s002.docx]

**Supplemental Figures**


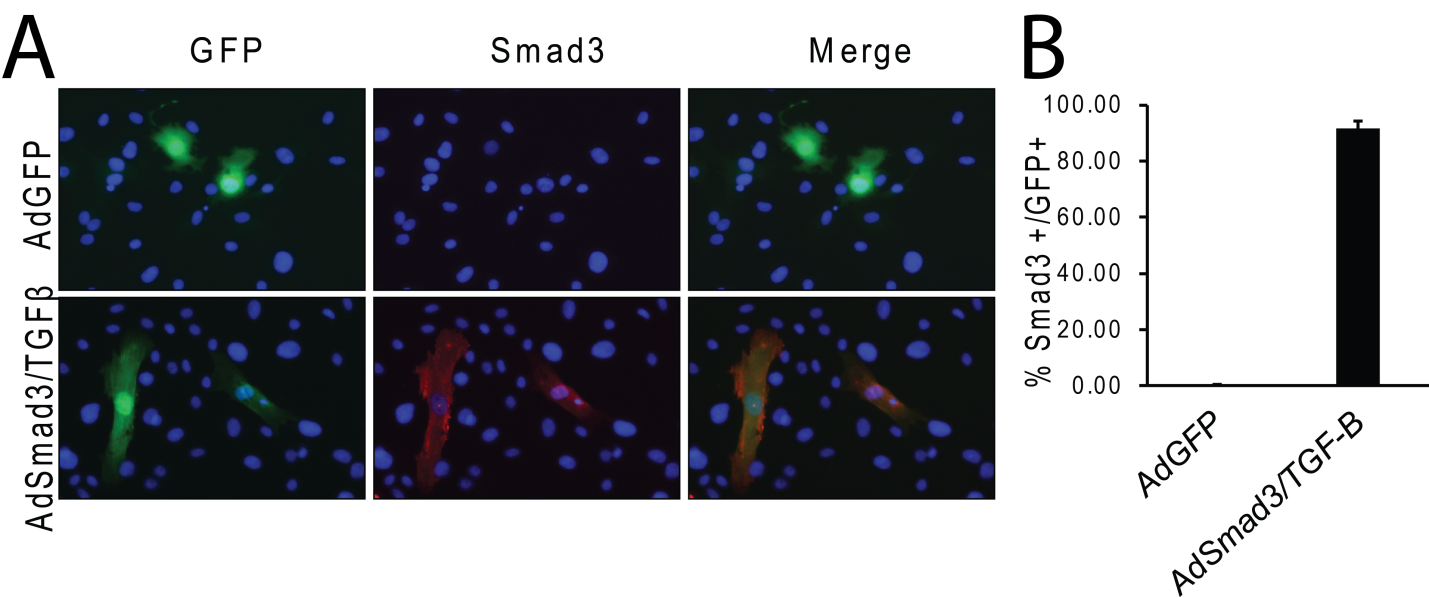


***Figure S1. Immunocytochemistry to detect Smad3 overexpression in AdSmad3-infected SMCs***

Rat vascular SMCs were infected with AdGFP or AdSmad3 and treated with TGF-β (5 ng/ml) for 24 hrs. Cells were fixed and subjected to immunostaining for Smad3 as described in detail in Materials and Methods. Smad3 positive cells were quantified as percent of GFP fluorescent cells. *P<0.05 compared to AdGFP, n=3.

***
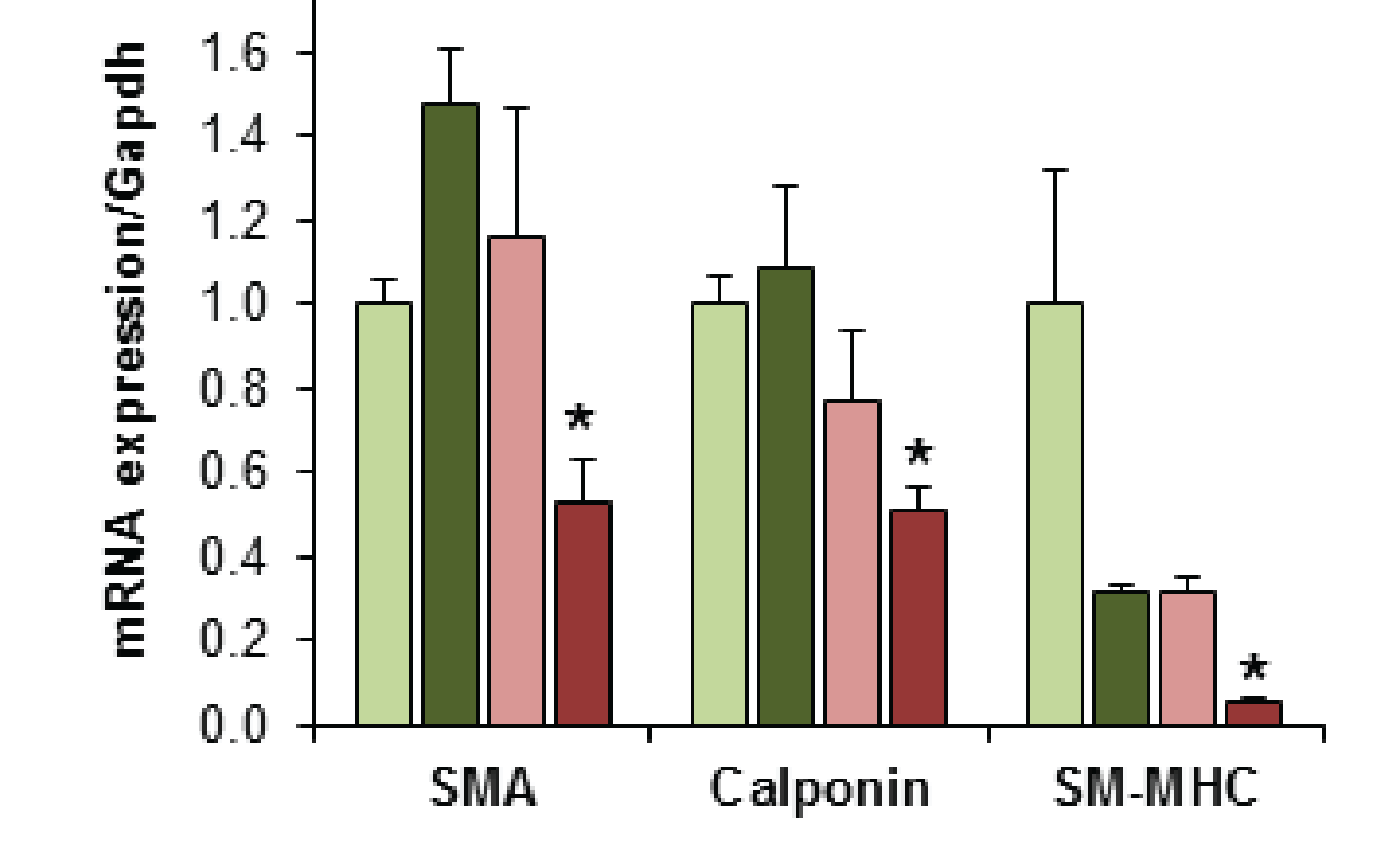
***

***Figure S2. Evaluation of gene expression of SMC markers regulated by AdSmad3/TGF-β treatment***

Rat vascular SMCs were infected with AdSmad3 and treated with TGF-β (5 ng/ml) for 24 hr (red). Controls were AdGFP (light green), AdGFP+ TGF-β (dark green) and AdSmad3 (pink). qRT-PCR was performed to evaluate gene expression of three SMC markers. *P<.05, compared to AdGFP; n=3.


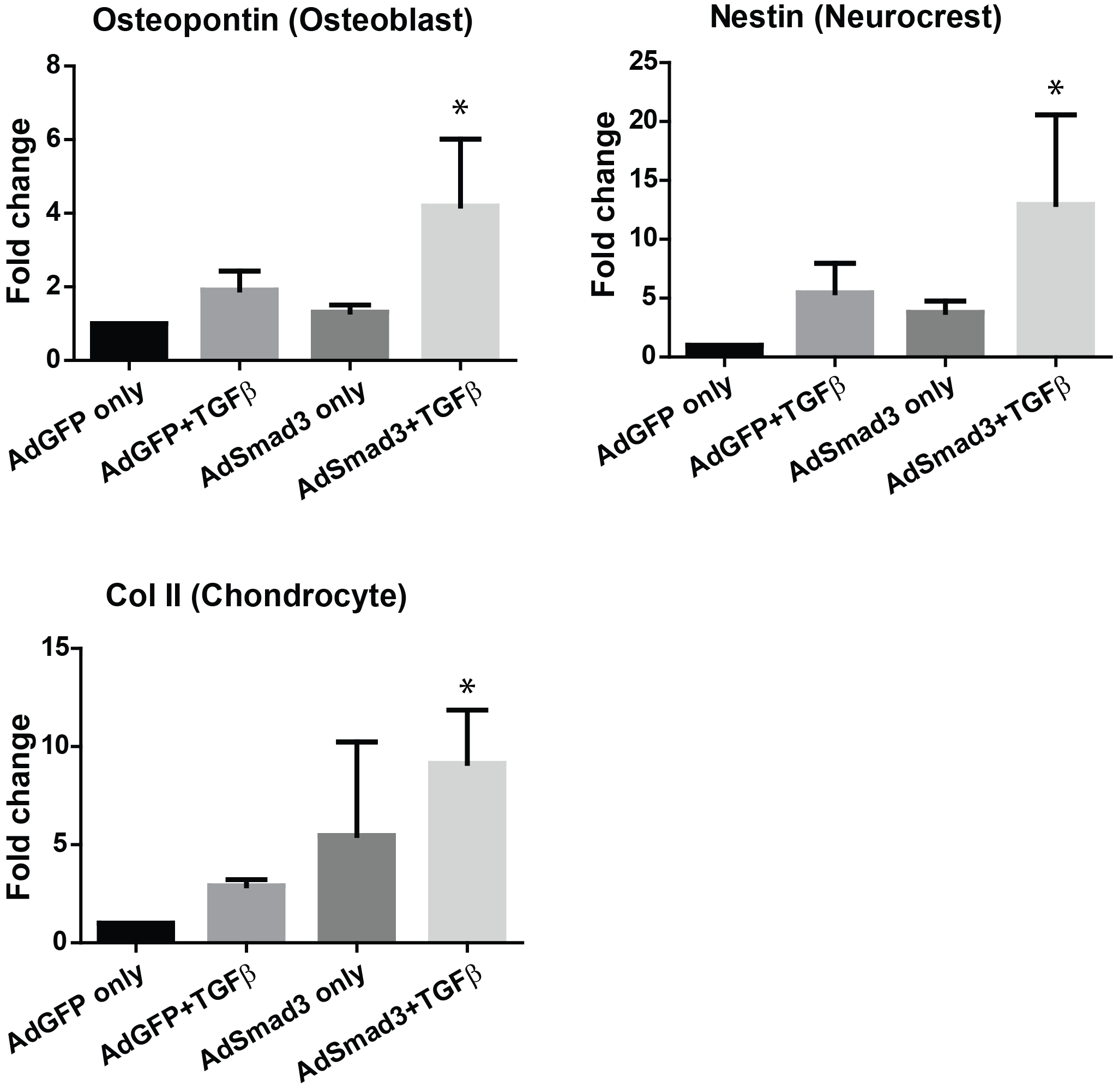


***Figure S3. AdSmad3/TGF-β treatment stimulates expression of chondrocyte, neurocrest, and osteopontin lineage markers***

Rat vascular SMCs were infected with AdSmad3 and treated with TGF-β (5 ng/ml) for 24 hr. qRT-PCR was performed to evaluate gene expression of collagen type II, nestin, and osteopontin, which are established markers for chodrocytes, neurocrest cells, and osteoblasts, respectively. Each bar represents a mean ± SD (n=3). * P<0.05, compared to AdGFP control.

***
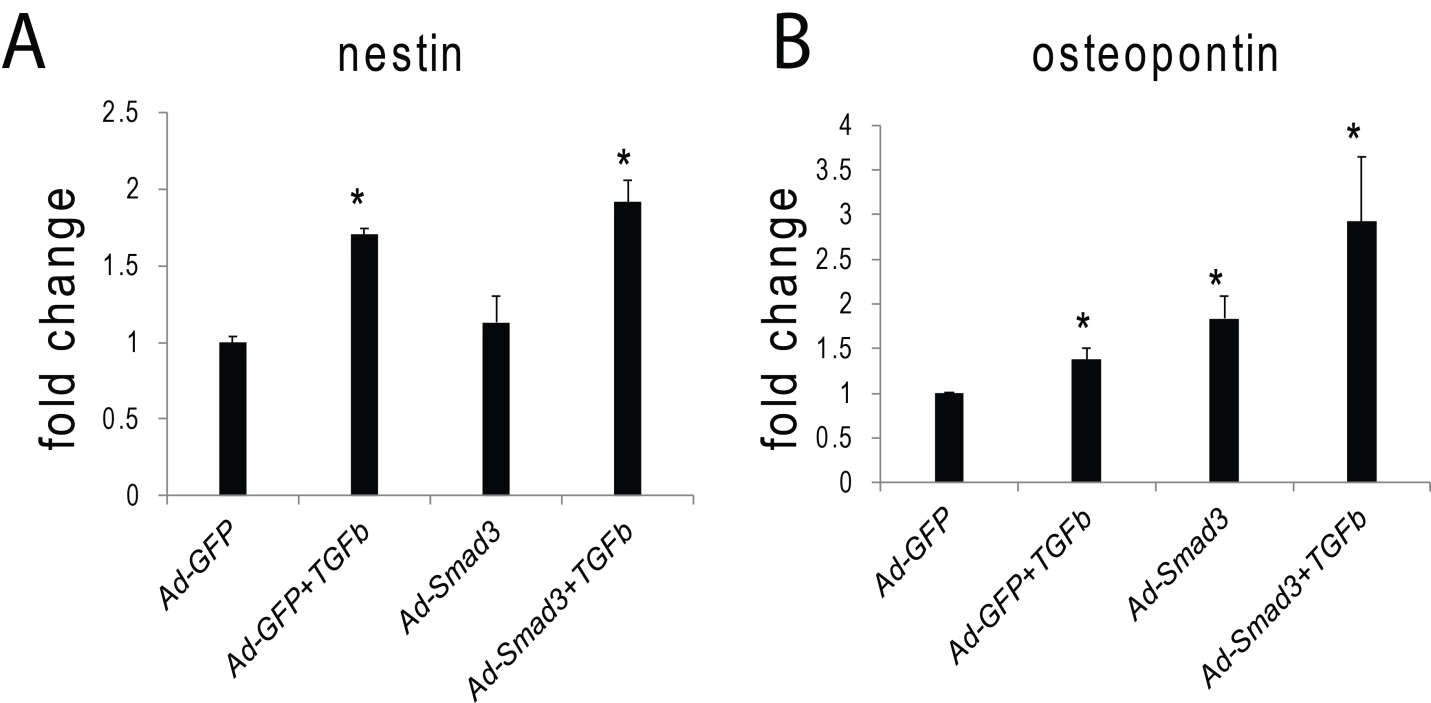
***

***Figure S4. AdSmad3/TGF-β-conditioned media stimulates expression of osteopontin and nestin in naïve SMCs***

Rat vascular SMCs were infected with AdSmad3 and treated with TGF-β (5 ng/ml) for 48 hr. Controls were AdGFP, AdGFP+ TGF-β and AdSmad3. Conditioned media collected from those cultures were added to naïve SMCs and incubated for 24h. qRT-PCR was then performed to evaluate gene expression of osteopontin and nestin. *P<.05, compared to AdGFP; n=4.
